# Inhibition abilities and functional brain connectivity in school-aged term-born and preterm-born children

**DOI:** 10.1038/s41390-024-03241-0

**Published:** 2024-06-19

**Authors:** Vera Disselhoff, Andras Jakab, Beatrice Latal, Barbara Schnider, Flavia M. Wehrle, Cornelia F. Hagmann, the EpoKids Research Group, Ulrike Held, Ruth Tuura O’Gorman, Jean-Claude Fauchère, Petra Hüppi

**Affiliations:** 1 Department of Neonatology and Pediatric Intensive Care, University Children’s Hospital Zurich, Zurich, Switzerland; 2 Children’s Research Center, University Children’s Hospital Zurich, Zurich, Switzerland; 3 Centre for MR Research, University Children’s Hospital Zurich, Zurich, Switzerland; 4 Neuroscience Center Zurich, University of Zurich, Zurich, Switzerland; 5 University of Zurich, Zurich, Switzerland; 6 Child Development Center, University Children’s Hospital Zurich, Zurich, Switzerland; 7 Department of Biostatistics at Epidemiology, Biostatistics and Prevention Institute, University of Zurich, Zurich, Switzerland; 8 Newborn Research, Department of Neonatology, University Hospital Zurich, Zurich, Switzerland; 9 Division of Development and Growth, Department of Woman, Child and Adolescent, University Hospitals of Geneva, Geneva, Switzerland

## Abstract

**Background:**

Inhibition abilities are known to have impact on self-regulation, behavior, and academic success, and they are frequently impaired in children born preterm. We investigated the possible contributions of resting-state functional brain connectivity to inhibition following preterm birth.

**Methods:**

Forty-four preterm and 59 term-born participants aged 8–13 years were administered two inhibition tasks and resting-state functional MRI was performed. Functional connectivity (FC) networks were compared between groups using network-based statistics. Associations of FCNs and inhibition abilities were investigated through multivariate linear regression models accounting for the interaction between birth status and inhibition.

**Results:**

NBS revealed weaker FC in children born preterm compared to term-born peers in connections between motor and supplementary motor regions, frontal lobe, precuneus, and insula. Irrespective of birth status, connections between the cerebellum, frontal, and occipital lobes and inter-lobar, subcortical, intra-hemispheric long-range connections were positively correlated with one of the two inhibition tasks.

**Conclusions:**

Preterm birth results in long-term alterations of FC at network level but these FCN alterations do not specifically account for inhibition problems in children born very preterm.

**Impact:**

- Irrespective of birth status, significant associations were found between the subdomain of response inhibition and functional connectivity in some subnetworks.
- A group comparisons of functional brain connectivity measured by rsfMRI in school-aged children born very preterm and at term.
- The investigation of network-level functional connectivity at rest does not appear adequate to explain differences in inhibition abilities between children born very preterm and at term, hence other imaging techniques might be more suited to explore the underlying neural mechanisms of inhibition abilities in school-aged children born very preterm.

## Introduction

Children born very preterm show an increased risk for impairments on various behavioral, academic, and cognitive domains.^1,2^ A set of higher-order cognitive processes fundamental for the effortful pursuit of a deliberately set goal, namely executive functions, is among the abilities frequently impaired.^3,4^ Research particularly focuses on inhibition abilities as they form an important component of executive functioning.^5–9^ Inhibition abilities consist of two aspects of inhibitory control: response inhibition and interference control,^9–12^ and strongly affect emotional regulation,^13^ (social) behavior,^14^ and long-term academic success.^15,16^ In individuals born very preterm, there is inconsistent evidence for either inhibition deficits,^17–36^ unimpaired inhibition abilities,^17,19,23,25,37–41^ or a possible catch-up to the abilities of typically-developing peers at middle school age.^29–32,42–49^ (for a comprehensive review, see ref. ^50^). In a previous study in preterm-born children at school age, we detected group differences in one subdomain of inhibition abilities, namely, in response inhibition, but neither in interference control nor overall inhibition abilities.^51^ This is in line with existing research.^33,42,45,46,52–54^ To better understand the complexity of inhibition problems in children born very preterm, the exploration of their neural correlates is indispensable. To find these neural correlates, network-level analysis of structural and functional brain connectivity offers valuable insights.^55^ We have shown that distributed structural parietal, cerebellar and subcortical connections are positively associated with inhibition abilities in both children born very preterm and in term-born children. However, group differences in inhibition abilities were not explained by different strengths of these structural connections.^51^ In typically developing individuals, meta-analytic evidence shows functional brain connectivity in a large-scale distributed brain network to be associated with inhibition abilities.^56^ FMRI at resting state (rsfMRI) assesses brain activity in the absence of deliberate activity or external stimulation, allowing the description of intrinsic functional brain networks showing synchronous, spontaneous neuronal activity.^57^ Interestingly, brain activity at rest has been shown to mirror task-induced activity patterns.^58^ Furthermore, resting-state brain activity has been reported to predict behavioral performance and related neural activity.^59,60^ Only a few studies investigated the effects of preterm birth on resting state functional connectivity (rsFC) in school-aged children.^61–64^ and very little literature exists on the potential association of rsFC with inhibition abilities in this population.^65^ Our aims were to compare rsFC between children born very preterm and at term, and to investigate the association of rsFC with inhibition abilities in middle-school agers born very preterm and at term. We hypothesized that rsFC is altered in children born very preterm compared to term-born children and can be associated with inhibition abilities.

## Materials and methods

### Participants

The EpoKids study is an ongoing prospective follow-up research project studying the long-term neuroprotective effect of erythropoietin (Epo) on executive functions in children born very preterm. Its recruitment procedure has been previously reported.^66^ While the original cohort includes children born between 2005 and 2012, the present study reports on children born between 2005 and 2009. A control group of term-born children were recruited through flyers, social media, or as friends/siblings of the very preterm-born participants. Inclusion criteria were birth at term in the absence of any neonatal complications, and no neurodevelopmental or neurologic disease at present or in the past.

### Study procedure

Neurodevelopmental assessments took place at the Child Development Center at the University Children’s Hospital Zurich (exception: seven assessments in Geneva; see “Results” for a detailed description) between July 2017 and September 2019. The assessment comprised an extensive neuropsychological test battery (approx. 7 h), which was administered in a pseudo-randomized order. The cerebral MRI scans were conducted at the MR center of the University Children’s Hospital Zurich.

### Instruments and measures

Perinatal data of the participants born very preterm were obtained from the original RCT at the Department of Neonatology at the University Hospital Zurich. Socioeconomic status (SES) based on parental education was estimated using a six-point scale (1–6).^67^ The summed scores of both parents resulted in total SES scores ranging from 2 to 12 in which higher values reflect higher SES.

### Neurodevelopmental assessment

IQ was estimated with four subtests of the Wechsler Intelligence Scale for Children^68^: block design, similarities, digit span forward/backward, and coding.^69^ Fine motor abilities were assessed with the pegboard task of the Zurich Neuromotor Assessment (ZNA).^70^

Detailed methods on the assessment of inhibition abilities in this cohort have been reported previously.^51^ Two standardized tasks were administered in random order examining the key functions of inhibition abilities: (1) The computerized stop signal paradigm assesses response inhibition by means of the stop signal reaction time (SSRT).^71^ (2) The D-KEFS’ Color-Word Interference Test (Stroop effect^72^) adjusted for individual processing speed^73^ assesses interference control. The results of both inhibition tasks were Fisher z-transformed using the mean and standard deviation of the control group to obtain equal scaling of the different tasks. *Z*-scores were combined in a single composite score reflecting overall inhibition abilities.

### Magnetic resonance imaging

Cerebral MRI was performed on a 3 T GE MR750 scanner using an 8-channel receive-only head coil. RsfMRI was acquired with a T2*-weighted EPI sequence, TE = 32 ms, TR = 1925 ms, flip angle = 74°, field of view = 27.8 cm, matrix = 64 × 64, voxel size = 4.3 × 4.3 mm, slice thickness = 3.6 mm, slice gap = 0 mm. During the rsfMRI sequence, participants were instructed to keep their eyes closed. Structural MRI was performed using T1-weighted 3D Spoiled Gradient Recalled (SPGR) sequence, TE = 5 ms, TR = 11 ms, inversion time = 600 ms, flip angle = 8°, field of view = 25.6 cm, reconstruction matrix = 256 × 256, voxel size = 1 × 1 mm, slice thickness = 1 mm.

### Resting-state pre-processing for functional connectivity analysis

For the processing of fMRI data, we utilized an in-house developed script in Bash language that wrapped functions from commonly used software packages (Digital Supplement). 3D-SPGR data were skull-stripped using the bet function in the FSL software.^74^ and the resulting skull-stripped brain images were visually controlled for possible masking errors, which were then corrected manually. Next, we created subject-specific anatomical masks in native fMRI space for calculating the time courses of various nuisance signals. The first step in this process was the co-registration of the temporally averaged, unprocessed fMRI dataset with the skull-stripped 3D-SPGR of the same subject using a 12-dof affine transformation in FSL (flirt command). Next, the 3D-SPGR image was linearly (flirt) and non-linearly co-registered (reg_f3d command in the NIFTYREG software library (based on the algorithm described in ref. ^75^)) with the corresponding T1-weighted anatomical template in the Automated Anatomical Labeling v.3. (AAL) atlas space, which step resulted in a deformation field that maps fMRI data to standard space. The inverse of the final transformation was calculated in order to transform atlas-derived anatomical region masks to the native fMRI space.

The following fMRI processing steps were carried out in the native space of the fMRI data. After slice-timing correction with the slicetimer command, we corrected spurious subject head motion with the fsl_motion_outlier command in FSL. Furthermore, image quality assessment was done visually on the mean fMRI image (native space), as well as by quantifying the mean framewise displacement (FD) value. Six movement parameters, three translations and three rotations, their first temporal derivative and the frame-wise displacement time course (FD) were saved as a column vectors. We exported the global signal and its first derivative based on a whole-brain mask. Next, voxels with high signal variability (noise voxels) were estimated by taking the voxels corresponding to the top 1 percentile value of the temporal standard deviation image. To clean the fMRI images from non-neuronal sources of signal variability, we retained the residuals after first-level regression comprising nuisance time courses using the 3dTproject command in the AFNI software.^76^ Nuisance signal regression was based on the CompCor^77^ method, during which the first three principal components of the anatomically defined time-courses were entered into the confound regression procedure in addition to the movement and global signal time courses. Anatomically defined time-courses were based on the native space masks covering the white matter and cerebro-spinal fluid spaces, of which masks were taken from the AAL atlas. Nuisance signal regression, spatial smoothing with a 5 mm Gaussian kernel and temporal band-pass filtering retaining frequencies 0.001–0.1 Hz were carried out in a single step with 3dTproject in AFNI. For group-level statistical analysis and functional connectivity calculation, the processed fMRI images were transformed and resampled to standard template with the nonlinear registration procedure described in this section.

The AAL labels were used to calculate time-courses for each of the 120 structures.^78,79^ Functional connectivity (FC) matrices were calculated as Pearson product–moment correlation coefficients between each time-course and matrices were Fisher *z*-transformed in the next step using the 3dNetCorr command in AFNI.

### Statistical methods

#### Demographic, neurodevelopmental, and cognitive data

Mean (*M*) and standard deviation (SD) are reported for the continuous variables and numbers and percentages of total for the categorical variables. Independent samples *t* test or Chi-squared test, as appropriate, were used to compare demographic and neurodevelopmental data between study groups. Using linear regression, the effect of preterm birth on inhibition abilities was explored while adjusting for age at assessment, sex, and SES to account for potential confounding bias. The exclusion of study cases from the model due to missing information on (one of) the parents’ education (*n* = 8) was avoided through single imputation applied for missing SES data. As the study is currently ongoing, continued blinding of the EpoKids study team was ensured by encoding the assignment at birth to either the intervention or placebo arm as “intervention 1” vs. “intervention 2” by the staff of the original trial. When neurodevelopmental outcomes were compared between the treatment groups to address potential intervention effects, no differences were found for IQ estimate, stop signal reaction time (SSRT), and Color Word inhibition time (all *p* > 0.05). Thus, when statistical analyses were conducted on the cognitive data, the treatment arm was not further taken into consideration and data of all very preterm participants were pooled. Statistical analyses were performed using *R* statistical software, Version 3.5.3.^80–84^ *p* Values <0.05 were considered statistically significant.

#### Statistical analysis of functional brain networks

Hypothesis tests on functional brain connectivity networks were based on the network-based statistics (NBS) described by Zalesky et al.^85^ implemented in the NBS Toolbox for Matlab R2014. NBS tackles the problem of multiple comparisons in tests on connectivity networks by estimating statistical significance for subsets of mutually connected network nodes in topological space.^86^ As NBS uses permutation test to build up the sample distribution, it can be applied to smaller study groups without assuming normality. In NBS, a hypothesis test is carried out for the empirically determined network components by comparing their extent with the proportion of permutations yielding a component with equal or greater size, correcting for the family-wise error rate at cluster level with *p* < 0.05. We chose a threshold *t*-score of 2.5 in our tests. Since fixed thresholding of connectivity matrices may bias the analysis of network organization in patient–control studies, we decided to carry out the NBS on the unthresholded networks. As our study is a between-group study, we first tested if global functional connectivity is different between the preterm and control children. We first tested global network differences between preterm and term-born children by averaging the functional connectivity value in all non-negative edges of unthresholded weighted networks and tested very preterm/term-born group differences using a two-sided Student’s *t* test. If these differences are significant, the global (mean) connectivity is included as a covariate in the statistical models.

We carried out three experiments using NBS. First, to reveal the functional connectivity differences associated with birth status, the functional brain connectivity networks were analyzed in NBS: in this model, sex and age at assessment were included (Table 1). The connectomes included only positive edges to avoid ambiguities related to the interpretation of anti-correlations after global signal regression.

**Table 1:** Models used in the analysis: preterm vs. control comparison.

	Preterms	Controls	Age at MRI	Sex	SES
Subj 1.	1	0	8.4	0	6
Subj 2.	0	1	7.6	1	5
…					
Contrast	−1	1	0	0	0

Design and contrast matrix for preterm vs. control comparison.

Second, we used the following analysis strategy to test whether neural correlates of inhibition abilities are different between very preterm and term-born children. We assessed the correlation of inhibition abilities using a multivariate linear regression model in NBS. These models were controlled for SES, sex, age at assessment, and group × inhibition interaction. We tested whether the very preterm/term-born grouping variable (birth status) interacted with the correlation between inhibition abilities and structural connectivity by performing a statistical test with continuous covariate interaction (contrast and design matrix: Table 2). Next, if these models were not significant (therefore, the slope of inhibition vs. FC was not different in any edge of the network between preterm and term-born children), we further evaluated a model using no interaction term (Table 3).

**Table 2:** Models used in the analysis: testing the interaction of birth status on the link between functional connectivity and inhibition, using continuous covariate interaction (categorical variable^a^ cognitive score).

	Preterm^a^	Controls^a^	Preterms	Controls	Age at MRI	Sex	SES
	Cognitive score^a^	Cognitive score^a^					
Subj 1.	−2.1	0	1	0	8.4	0	6
Subj 2.	0	−1.8	0	1	7.6	1	5
…							
Contrast 1	1	−1	0	0	0	0	0
Contrast 2	−1	1	0	0	0	0	0

^a^Inhibition score, response inhibition, or interference control, *Z*-score.

**Table 3:** Models used in the analysis: testing the association of inhibition score on functional connectivity, irrespective of the birth status.

	Preterms	Controls	Cognitive score^a^	Age at MRI	Sex	SES
Subj 1.	1	0	−2.1	8.4	0	6
Subj 2.	0	1	−1.8	7.6	1	5
…						
Contrast 1	0	0	1	0	0	0
Contrast 2	0	0	−1	0	0	0

Design and contrast matrix.

^a^Inhibition score, response inhibition, or interference control, *Z*-score.

The functional connectivity analysis results were presented as three-dimensional graph visualizations, which represented below *p*-threshold connection pairs surviving multiple comparison correction, as defined at the cluster level in NBS. These brain networks were visualized with the BrainNet Viewer for Matlab R2014. Circular plots of below *p*-threshold connection pairs were visualized with the circularGraph tool for Matlab R2014.^87^

## Results

### Sample characteristics

100 children and their parents out of 180 eligible participants, agreed to participate in the EpoKids study (55.6%). Participants and non-participants did not differ in terms of gestational age, birth weight, neonatal complications (BPD, NEC, ROP, major brain lesions), and Bayley scores^88^ at 2 years of age (all *p* > 0.05). The SES of participants’ parents was higher than of non-participants (*p* = 0.004). No MRI data was available for seven participants from Geneva, and for fourteen families who completed questionnaires only. These 21 participants were, thus, not further considered. 12 very preterm and 9 term-born participants were excluded from the current analyses as they were not administered one or both inhibition tasks due to technical issues or indication of fatigue. This resulted in 67 very preterm and 69 term-born participants. Participants with and without complete inhibition data were compared with regard to sex, age at assessment, SES, and estimated IQ to test for a potential selection bias that did not reveal any differences (all *p* > 0.05). Table 4 summarizes demographic, socioeconomic, and perinatal data of all participants of the prospective study. While SES was higher in families of term-born participants, no group differences were found for sex and age at assessment.

**Table 4:** Demographic, socioeconomic, and perinatal data of participants.

	Very preterm born *n* = 67			Term born *n* = 69			*p*^a^
Demographic and socioeconomic data							
Female, *n* (%)	28 (41.8)			32 (46.4)			0.72
	***M***	**SD**	**Range**	***M***	**SD**	**Range**	
Age at assessment (in years)	10.8	1.2	[8.8–13.4]	11.1	1.3	[8.8–13.5]	0.18
Socioeconomic status^b^	7.9	2.0	[4–12]	9.7	2.1	[6–12]	<0.001
Perinatal data							
Gestational age (in weeks)	29.3	1.7	[26.0–31.7]	39.6 1.1		[37.3–42.0]	
Birth weight (in g)	1210	317	[570–2020]	3470 448		[2370–4410]	
Moderate or severe BPD, *n* (%)	7 (10.4)			–			
ROP ≥ grade 3, *n* (%)	0 (0)			–			
NEC, *n* (%)	3 (4.5)			–			
Major brain lesions, *n* (%)	2 (3)			–			

*M* mean, *SD* standard deviation, *BPD* bronchopulmonary dysplasia, *ROP* retinopathy of prematurity, *NEC* necrotizing enterocolitis.

^a^Independent samples *t* tests.

^b^Possible range for total SES scores: 2–12. Missing SES data: *n* = 8.

### Neurodevelopmental assessment

The estimated IQ was lower in very preterm than in term-born children (*p* < 0.001). Differences in fine motor abilities were not explained by preterm birth when adjusting for age at assessment, sex, and SES (*β* = 0.1, 95% CI [−0.74, 1.24], *p* = 0.62; adjusted *R*^2^ = 0.07, *p* < 0.016). The following results on group comparisons of inhibition abilities have been reported previously^51^: the *Z*-score of the overall inhibition composite score was lower in the very preterm than in the term-born group (*p* = 0.007) and of the two inhibition measures, only the *Z*-score of the SSRT was lower in the very preterm-born children (*p* = 0.001). When adjusting for age at assessment, sex and SES, differences in the overall inhibition composite score were not explained by preterm birth (*β* = −0.13, 95% CI [−0.30, 0.04], *p* = 0.13; adjusted *R*^2^ = 0.19, *p* < 0.001), but differences in the SSRT remained significant (*β* = 0.25, 95% CI [−0.43, −0.08], *p* = 0.006; adjusted *R*^2^ = 0.11, *p* < 0.001). Differences in Color Word inhibition time were not explained by preterm birth when adjusting for age at assessment, sex, and SES (*β* = 0.07, 95% CI [−0.11, 0.24], *p* = 0.46; adjusted *R*^2^ = 0.15, *p* < 0.001). Table 5 summarizes the neurodevelopmental data of the participants included in the study.

**Table 5:** Neurodevelopmental data of participants.

Cognitive and motor data	Very preterm born *n* = 67			Term born *n* = 69			*p*^a^
	*M*	SD	Range	*M*	SD	Range	
IQ estimate	102	16.5	[67.1 to 138]	115	13.7	[83.7 to 152]	<0.001
Fine motor abilities (in s)	18.3	2.6	[13.5, 28.5]	17.4	2.3	[13.0, 24.0]	0.036
Inhibition composite score^b^	−0.4	0.8	[−2.2 to 1.7]	0.0	0.8	[−2.3 to 1.5]	0.007
SSRT^b^	−0.6	1.1	[−3.5 to 3.1]	0.0	1.0	[−2.9 to 1.9]	0.001
Color Word inhibition time (2 s)	−0.1	0.9	[−2.8 to 1.5]	0.0	1.0	[−3.1 to 1.5]	0.5

*M* mean, *SD* standard deviation.

^a^Independent samples *t* tests.

^b^*Z*-scores calculated with *M* and SD of term-born group.

#### Availability and quality of rsfMRI data

For 12 very preterm and 5 term-born children, there were no rsfMRI data available as they refused (full) MRI assessment. Further, the rsfMRI data of 11 very preterm and 5 term-born children was excluded from analyses due to poor data quality (i.e., susceptibility artifacts caused by orthodontic appliances or strong movement artifacts, meaning mean FD > 0.5 mm or determined by visual inspection of the mean image). Thus, for a subsample of 44 children born very preterm and 59 children born at term, rsfMRI data were available and included into the functional connectivity analysis. The characteristics of participants with and without rsfMRI data were compared regarding birth status, sex, age at assessment, SES, and estimated IQ. Estimated IQ, age at assessment, sex distribution, and SES were similar between groups (all *p* > 0.05).

### Group comparison of functional connectivity on network level

Mean functional connectivity was not different between the children born very preterm and at term (*p* = 0.538). Between-group effects were measured using NBS on the positive edges of the unthresholded, weighted networks.

NBS analysis revealed that functional connectivity was weaker in very preterm-born children in a subnetwork consisting of 26 network edges shared by 21 nodes (*p*-minimum = 0.05, *t*-maximum: 3.519). This network consisted of inter-lobar long-range connections mainly connecting the motor and supplementary motor regions, frontal lobe, precuneus and insula. The nodes with the highest number of significant edges were the following: right supplementary motor area, right cerebellum lobule VII/b, left postcentral gyrus, right precuneus, left middle temporal pole—middle part, left putamen, left precentral gyrus, right Heschl gyrus, left inferior parietal lobule, right Rolandic operculum.

### Functional connectomic correlates of inhibition abilities in children born very preterm and at term

**Fig. 1:**
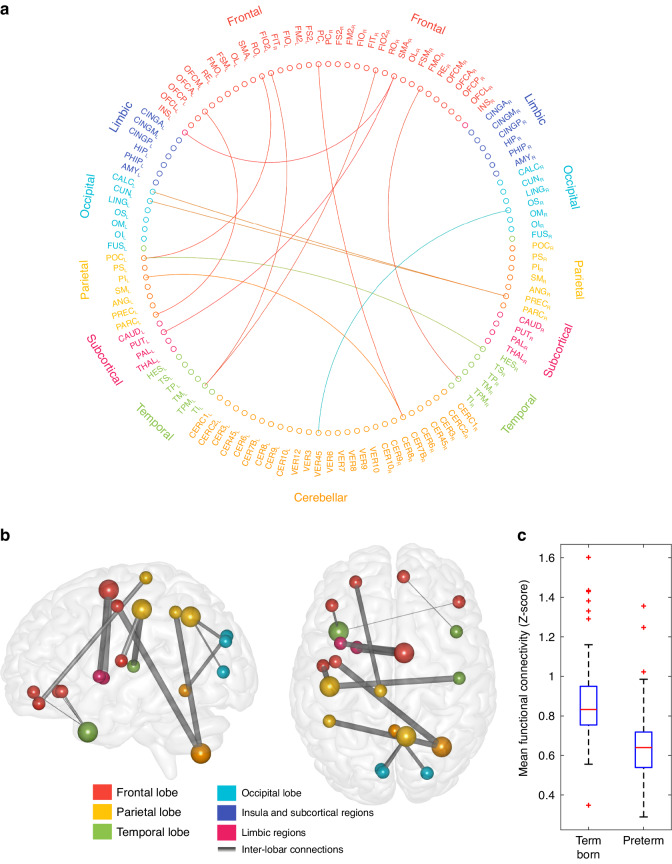
Differences between the functional connectivity network of very preterm and term-born children. **a** Circular plot depicting the edges where the correlation was significant, and **b** 3D graph showing the functional connections significantly lower in very preterm compared to term-born children. **c** Boxplot showing the mean functional connectivity differences in this subnetwork in very preterm compared to term-born children.

The inhibition composite score and interference control score were not correlated with functional connectivity at the network level and the models with an interaction effect of birth status on the functional connectivity and total inhibition or interference control were also non-significant (all *p* > 0.05) (Fig. 1).

**Fig. 2:**
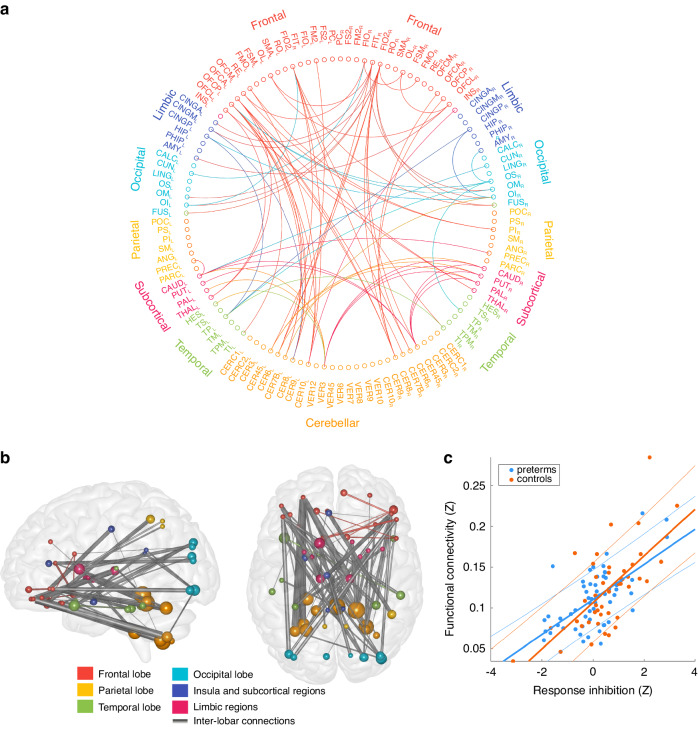
Correlation of functional connectivity with response inhibition in very preterm and term-born children. **a** Circular plot depicting the edges where the correlation was significant, **b** visualization of the sub-network, **c** linear regression plot showing very preterm and term-born children separately.

For the response inhibition score, the model using continuous covariate interactions revealed no significant interaction effect of birth status on the functional connectivity and response inhibition score. However, in a post hoc NBS analysis without the interaction term in the model, we found a subnetwork in which functional connectivity was positively correlated with the (*p*-minimum = 0.046, *t*-maximum = 4.08). This network consisted of 34 nodes sharing 77 network edges, the latter corresponding to 2.3% of the total non-negative edges in the whole-brain connectivity network. This network was characterized by antero-posteriorly running long-range connections between the cerebellum and the frontal and temporal lobes or further inter-lobar, intra-hemispheric long-range connections (Fig. 2a, b). The nodes with the highest number of significant edges were the following: lobule IV and V of both cerebelli, lobule III. of the vermis, lobule XVIII of the both cerebelli, lobule X of the left cerebellum, lobule VI of the left cerebellum, right inferior occipital gyrus, left caudate, left superior temporal pole, lateral part of the right orbitofrontal cortex, right middle and superior occipital gyrus, left inferior occipital gyrus, right fusiform gyrus, left and right thalamus.

Visual inspection of the regression lines show that in the network in which functional connectivity was significantly correlated with response inhibition scores, the slope of a linear function fitted on the functional connectivity values did not show considerable differences between children born very preterm and at term (Fig. 2c), confirming the negative finding in the model with the interaction term.

## Discussion

This is one of the first studies investigating the association between inhibition abilities and functional connectivity on network level at rest in middle school-aged children born very preterm and at term. Overall, inhibition abilities were comparable in both study groups, although differences were observed for the subtest response inhibition. Functional brain connectivity was similar between children born very preterm and term-born peers. However, NBS revealed a subnetwork of weaker functional connectivity in children born very preterm compared to term-born peers, particularly in connections between the motor and supplementary motor regions, the frontal lobe, precuneus, and insula. Irrespective of birth status, significant associations were found between the subdomain of response inhibition and functional connectivity in a subnetwork predominantly consisting of antero-posteriorly running long-range connections between the cerebellum and the frontal and occipital lobes in addition to subcortical and further inter-lobar, intra-hemispheric long-range connections.

Several studies set out to investigate whether very preterm birth has detrimental effects on the development of intrinsic brain networks in neonate infants as the emergence of RSNs revealed by fMRI largely seems to take place during the third trimester of gestation in the period of rapid neural growth.^89–91^ Whereas one study reported a similar repertoire of RSNs to be present in a cohort of very preterm infants at term-equivalent age as in term-born infants,^89^ there are significant differences in the presence of connections in infants born prematurely vs. those term born.^92^ Importantly, across several brain regions included in well-established RSNs (Brodmann areas, anterior cingulate, anterior insula, sensory/motor network of the medial and dorsal superior frontal gyrus bilaterally, left supplementary motor area, left angular gyrus, right fusiform gyrus, sensorimotor cortex, thalamus, brainstem regions), weaker functional connectivity has been reported in infants born very preterm at term equivalent age.^92–96^ The present study is amongst few others to report on group comparisons of functional brain connectivity measured by rsfMRI in school-aged children born very preterm and at term. Partly overlapping with the mentioned studies in younger age groups, we found significantly weaker functional connectivity in school-aged children born very preterm compared to term-born peers in the motor and supplementary motor regions, frontal lobe, precuneus and insula. Significant alterations of white matter microstructure have been identified in the present cohort.^51^ This is of particular interest as alterations of resting-state functional brain connectivity in infants born prematurely have been associated with early aberrant microstructural development.^97–99^ While the current study only found evidence for decreased functional connectivity in children born very preterm, previous studies in school-agers identified co-occurring patterns of increased and decreased functional connectivity in the very preterm group compared to term-born peers.^61^

Precisely, stronger connectivity was identified between the sensorimotor network and both the salient network and the central executive network in the very preterm group.^61^ The “central executive network” includes dorsolateral prefrontal and parietal regions,^100^ while the “salience network” is focused in frontoinsular cortex, dorsal anterior cingulate cortex, and subcortical structures. Concurrently, weaker connectivity was identified between the sensorimotor network and both the visual network and the dorsal attention network, within the sensorimotor network and within the central executive network.^61^ Similarly, in preadolescent children born late preterm, rsfMRI revealed both decreased and increased functional connectivity compared to term-born peers: increased functional connectivity was observed within regions of the default mode, salience, and central-executive network decreased functional connectivity was observed within the right parahippocampal region.^62,63^ Decreased functional connectivity strength has commonly been construed as a consequence of long-lasting detrimental effects of preterm birth on the integrity of intrinsic brain network connectivity (e.g., ref. ^101^). Degnan et al. interpreted their finding of “hyperconnectivity” in preadolescents born late preterm as a failure to progress beyond rudimentarily distributed networks, thus, mirroring a disruption of normal synaptic pruning.^62,63^ Other authors suggested increased functional network connectivity in individuals born very preterm has been suggested to reflect an adaptive mechanism in order to compensate for early neurologic insult, i.e., engagement of alternate circuits, as a consequence of very preterm birth.^102^

It has been reported previously that brain activity at rest largely mirrors task-induced activity patterns.^58^ Moreover, resting-state brain activity has been shown to predict task performance and task-induced/related neural activity: studies in neonates born very preterm found cortical and amygdala functional brain connectivity at rest to be related to behavioral inhibition at 2 years.^103,104^ Recently, Wheelock et al.^65^ found poorer attention scores in 12-year-old children born very preterm to be associated with ventral attention, visual, and subcortical networks, whereas attention functioning was associated with between-network connectivity in the frontoparietal, cingulo-opercular, dorsal attention, salience and motor networks in children born at term children. In the present study, we did not confirm findings of aberrant associations of functional brain connectivity with inhibition abilities in children born very preterm compared to term-born peers when looking at resting state network connections. Irrespective of birth status, we found the subdomain of response inhibition to be associated with functional brain connectivity at rest in the cerebellum, frontal and temporal lobes, and further inter-lobar, intra-hemispheric long-range connections. These findings are partly in line with meta-analytic evidence accumulated from task-based fMRI studies in healthy adults: mapping activation patterns to a brain functional network atlas revealed the insula cortex, the right supplementary motor area, the bilateral superior temporal gyri, and the right inferior parietal lobule to be the core neural systems commonly involved in the process of response inhibition.^56^ Further, a study with former very preterm adults reported similar brain regions to be active during a response inhibition task.^105^ In the current study, we did not find an association between functional brain connectivity at rest and the performance on an interference control task. This is in contrast to the findings of the meta-analysis mentioned: evidence of studies using task-based fMRI was reported that the cognitive process of interference control involves the activation of the left supplementary motor area, the left inferior parietal lobule, the left precentral gyrus, the right insula, the right middle frontal gyrus, and bilateral inferior frontal gyri.^56^ To date, differences in data acquisition (e.g., rsfMRI vs. task-based fMRI studies), the granularity of data analyses (network-level, node or global-level) and the assessed population (e.g., typically developing children vs. children at different levels of prematurity vs. adults) limit comparability between studies. In the present study, only the subdomain of response inhibition—the domain in which very preterm children performed significantly lower compared to term-born peers—was significantly associated with functional brain connectivity at rest. Potentially, the inherent inter-subject variability which, across both study groups, was larger on the subdomain of response inhibition statistically facilitates the detection of both between-group differences in performance as well as correlations with functional brain connectivity. More studies in similar populations are needed to confirm the presented results and further investigate their implications.

### Limitations

Our study has several limitations. The inclusion criteria of the EpoKids study led to a sample of very preterm participants who had relatively high estimated IQ scores, came from a high socioeconomic background, and had few neonatal complications. The cohort, thus, may not be representative of the general population of school-aged children born very preterm.^106^ Possibly, it also explains why group differences in overall inhibition performance were rather small between children born very preterm and at term, even if they are partly statistically significant. Further, several participants had to be excluded from the analyses due to motion or dental braces artifacts. This resulted in a less well-balanced cohort eligible for the rsfMRI analysis with regard to birth status (significantly more term-born children) which may have impacted the representativeness of imaging results. Technically, we used a static measure of functional connectivity based on time course correlation. However, FC is dynamic in the human brain, as reflected by a switching between various states within which the FC appears to be static.^107–109^ This has been the subject of several more recent investigations, many of which found a link between the role of this switching behavior and cognitive processing and performance in neuropsychological tests.^110,111^

Generally, the comprehensive study protocol of the EpoKids study resulted in a reduced study cohort when compared to the number of children that were originally eligible for the present analyses. However, the sample size is comparable to other studies in this research field.^61,62,112–114^

## Conclusion

The present analyses show long-term detrimental effects of preterm birth on functional connectivity at network-level alongside comparable overall inhibition abilities in very preterm children at middle school age compared to term-born peers. However, the investigation of network-level functional connectivity at rest does not appear adequate to explain differences in inhibition abilities between children born very preterm and at term. Possibly, considering and combining graph theoretical parameters of structural connectivity (e.g., local and global efficiency, clustering coefficient, betweenness centrality) with measures of functional connectivity, will shed light on these issues in the future.

## Data Availability

Sensitive personal health-related data obtained during the prospective study cannot be shared publicly due to ethical restrictions on secondary use. By contacting the corresponding author, anonymized, post-processed research data, such as connectivity matrices in this manuscript, can be obtained. The fMRI processing script is available as a Digital Supplement.
